# Self-reported post-exertional fatigue in Gulf War veterans: roles of autonomic testing

**DOI:** 10.3389/fnins.2013.00269

**Published:** 2014-01-07

**Authors:** Mian Li, Changqing Xu, Wenguo Yao, Clare M. Mahan, Han K. Kang, Friedhelm Sandbrink, Ping Zhai, Pamela A. Karasik

**Affiliations:** ^1^Department of Veterans Affairs Medical Center, War-Related Illness and Injury Study CenterWashington, DC, USA; ^2^Neurology Service, Department of Veterans Affairs Medical CenterWashington, DC, USA; ^3^Research Service, Department of Veterans Affairs Medical CenterWashington, DC, USA; ^4^Cardiology Service, Department of Veterans Affairs Medical CenterWashington, DC, USA

**Keywords:** self-reported symptom, post-exertional fatigue, objective testing, autonomic, Gulf War veteran

## Abstract

To determine if objective evidence of autonomic dysfunction exists from a group of Gulf War veterans with self-reported post-exertional fatigue, we evaluated 16 Gulf War ill veterans and 12 Gulf War controls. Participants of the ill group had self- reported, unexplained chronic post-exertional fatigue and the illness symptoms had persisted for years until the current clinical study. The controls had no self-reported post-exertional fatigue either at the time of initial survey nor at the time of the current study. We intended to identify clinical autonomic disorders using autonomic and neurophysiologic testing in the clinical context. We compared the autonomic measures between the 2 groups on cardiovascular function at both baseline and head-up tilt, and sudomotor function. We identified 1 participant with orthostatic hypotension, 1 posture orthostatic tachycardia syndrome, 2 distal small fiber neuropathy, and 1 length dependent distal neuropathy affecting both large and small fiber in the ill group; whereas none of above definable diagnoses was noted in the controls. The ill group had a significantly higher baseline heart rate compared to controls. Compound autonomic scoring scale showed a significant higher score (95% CI of mean: 1.72–2.67) among ill group compared to controls (0.58–1.59). We conclude that objective autonomic testing is necessary for the evaluation of self-reported, unexplained post-exertional fatigue among some Gulf War veterans with multi-symptom illnesses. Our observation that ill veterans with self-reported post-exertional fatigue had objective autonomic measures that were worse than controls warrants validation in a larger clinical series.

## Introduction

About 7.7% of 11,441 survey respondents among a sample of 15,000 veterans after 1990–1991 deployment to Gulf War (GW) theater reported a variety of neurological symptoms in a population based study conducted in 1995 (Kang et al., 2000, 2002). A follow up survey in year 2005 on these GW veterans showed that neuromuscular multi-symptoms such as post-exertional fatigue, dizziness, and tremor remained as medically unexplained (Kang et al., 2003, 2009; Blanchard et al., 2006). Sophisticated neurophysiologic investigation in representative ill GW veterans showed that the unexplained neuromuscular complaints were not likely to be due to the primary muscle or neuromuscular junction abnormalities (Amato et al., 1997; Sharief et al., 2002). Large fiber neuropathy screening showed the same occurrence rate in these veterans compared to non-deployed Gulf era controls, suggesting the need for investigating autonomic nervous system because the vague neuromuscular symptoms were likely to correlate to the small somatic or autonomic nerve fibers rather than large fibers (Jamal et al., 1996; Davis et al., 2004; Rose et al., 2004).

In this study we compared the autonomic outcomes between 16 ill GW veterans with chronic post-exertional fatigue and 12 GW controls. All 28 study participants were deployed to GW theater during the period 1990–2002. Participants from the ill group had chronic post-exertional fatigue in the epidemiological survey conducted in 2005, and that many years later the post-exertional fatigue remained a chief complaint at the time of the current study conducted in 2011–2012. The controls denied post-exertional fatigue at the time of the 2005 survey and during the current study. In the clinical context we searched for the presence of relevant clinical autonomic disorders through objective autonomic testing and compared the group differences on measures of cardiovascular function at both baseline and head-up tilt, and sudomotor function.

## Material and methods

### Study participants

In the survey entitled “Health of US Veterans of 1991 Gulf War: a Follow-Up Survey in 10 years” conducted in year 2005, many deployed GW veterans reported chronic fatigue syndrome-like multi symptoms (Kang et al., 2009). Based on this survey we generated Ill (*n* = 200) and Control (*n* = 398) pools of potential study participants in Washington, DC and nearby states determined mainly upon their response to the survey question that inquired whether there was recurrent post-exertional fatigue lasting more than 24 h or chronic fatigue longer than 6 months since the deployment to the Gulf theater. We mailed 150 invitation letters to veterans from each of the above 2 pools, requesting participation in a clinician administered telephone interview to determine the participant eligibility for the on-site clinical study. Potential participants were chosen according to a stratified sampling plan, with strata defined by 3 age subgroups (<42-years old, 42–52, and 53–65). There are 61 (41%) respondents from the Ill pool and 42 (28%) controls who participated in the telephone interview. This telephone interview questionnaire had a total of 80 questions that are classified into 5 sections: (1) medical history, (2) autonomic symptoms, (3) neuromuscular fatigue, (4) unexplained tremor, and (5) irritable bowel. The study entry criteria for final Ill vs. Control group were determined upon either the presence for the Ill group, or absence for the Control group, of both (a) symptoms of post-exertional fatigue more than 24 h or chronic fatigue longer than 6 months self-reported at the 2005 survey and (b) that post-exertional fatigue lasting more than 24 h was the chief complaint at the time of telephone interview. The exclusion criteria for the final data analysis used in this report included any one or more of the following: (a) past or current diagnoses of psychotic disorders or major affective disorders (9 Ill and 2 Control respondents), (b) taking medications that cannot be withheld for 10 days prior to the autonomic testing (14 Ill and 5 Control respondents), (c) conditions that likely interfere with autonomic testing interpretation (13 Ill and 8 Control respondents), (d) inability or unwillingness to travel (5 Ill and 14 Control respondents), (e) those who do not meet study entry criteria (4 Ill and 3 Control respondents), and (f) conditions that likely explain the post-exertional fatigue (16 Ill respondents). Sixteen (26%) out of the above 61 Ill respondents and 12 (29%) out of above 42 Control respondents met both inclusion and exclusion criteria, thus constituted the final Ill and Control below, respectively.

### Measures and procedures

We performed objective clinical study on both Ill (*n* = 16; age range: 39–58 year-old) and Control (*n* = 12; age range: 40–64 year-old) at the neuromuscular unit of Veterans' Hospital at DC after the approval by Research and Development Committee/Institutional Review Board. On day 1 we administered autonomic nervous system related questionnaires, and conducted history and physical examinations, and trained participants to become familiar with testing procedures; on day 2 conventional large fiber nerve conduction studies (NCS) (Cadwell, Kennewick, WA), quantitative sensory testing [(QST) NTE-2 and Vibratron II, Physitemp Instrument, Clifton, NJ], and baseline autonomic testing; on day 3 quantitative sudomotor axon reflex test [(QSART) WR Electronics, Quantitative Sweat Measurement System, Stillwater, MN] and diagnostic Tilt-Table Test. Testers were not blinded to the subject groups. Two investigators of the study (ML and FS) are board certified in Clinical Neurophysiology, 1 (ML) board certified in Clinical Autonomic Disorder by United Council for Neurological Subspecialties, and 1 (PK) board certified in Cardiology.

All 28 participants received clinical history and physical examination and completed questionnaires regarding neuromuscular fatigue and autonomic symptoms. We obtained post-exertional Fatigue Severity Score (0, 1, 2, 3, 4, and 5 points) for each participant based on the response to the question asking for the late adverse effect of 30 min physical activity at 6–7 metabolic equivalents on participant's ability to perform daily routines 24 h later after such activity. Post-exertional Fatigue Severity Score was scored as 0 point if that answer was “None,” 1 for “Minimal,” 2 for “Moderate,” 3 for “Substantial,” 4 for “Severe,” and 5 for “Very Severe.” We performed NCS with repetitive nerve stimulation on 1 motor nerve and NCS on 1 sensory nerve from the dominant-hand side of the leg unless there was history of fracture or local skin disorder on this side, on each of the 28 participants, including 20 right and 8 left peroneal motor nerves as well as 21 right and 7 left sural nerves. If examination or NCS suggested neuromuscular disorders we then performed additional electrophysiological studies. Fourteen participants in the Ill group and 3 controls received concentric needle electromyography at one distal and one proximal leg muscle. The presence of both (a) symptoms or examination compatible with peripheral neuropathy and (b) an abnormal nerve conduction study were necessary for diagnosis of the length-dependent axonal large fiber neuropathy (Dyck et al., 1985). We used diagnostic definition of small fiber neuropathy (SFN) which requires both (a) either positive or negative symptoms of distal limbs with compatible abnormalities on QSART and (b) normal NCS (Stewart et al., 1992). Fourteen participants in Ill group and 11 Controls received NCS of at least one hand examining for carpal tunnel syndrome (CTS) and ulnar neuropathy. We performed QST for thermal and vibration threshold on index fingers and big toes of all 28 participants as an additional approach comparing the group differences regarding small or large nerve fiber function (Arezzo and Schaumburg, 1989). We used the procedure of “Method of Limits” to determine the thermal and vibration threshold (Arezzo, 1988).

Baseline cardiovascular autonomic testing included the blood pressure and heart rate at the end of supine resting for 5 min, active standing for 3 min, and passive head up tilt (HUT) at 70° for 5 min. We performed Heart Rate Variability to deep breathing (HRV_DB_)(CADWELL, Kennewick, WA) expressed as E/I ratio and Heart Rate Variability to Valsalva maneuver (HRV_VM_) as Valsalva ratio (Ravits, 1997). Beat-to-beat blood pressure and pulse rate were recorded continuously at a finger through Finometer Pro (FMG, Amsterdam, Netherlands) with simultaneous brachial blood pressure, heart rate, and pCO_2_ recording by an electronically controlled instrument (WelchAllen, Beaverton, Oregon). One participant in the Ill group declined the 5 min baseline HUT for a non-medical reason but completed all other baseline cardiovascular procedures, and other 27 participants completed all baseline autonomic procedures. About 24 h after the 5 min baseline HUT testing mentioned above, we used the 20 min diagnostic HUT at 70° on an electronically controlled tilt table (Midland Manufacturing, Columbia, SC) to assess the orthostatic intolerance. All 12 Controls completed the 20 min HUT. Three participants in the Ill group (*n* = 16) did not complete the 20 min tilt table test: 1 participant terminated HUT at 12 min due to persistent dizziness, 1 terminated at 6 min due to the near syncope symptoms with orthostatic hypotension, and 1 who declined baseline HUT also declined the 20 min HUT.

Composite Autonomic Scoring Scale (CASS) was used to compare the group difference in overall autonomic function (Low et al., 2004). We performed QSART using a total of 8 standard recording sites: forearms, proximal and distal lower legs, and feet (Low et al., 1983). We described the patterns of sudomotor abnormalities as: (a) the length dependent abnormality (a reduction in distal sweat volume to a level of 1/3 the size or less of the proximal lower leg), and (b) the patchy abnormality (a reduction of sweat volume below the lower limit on 2 or more of the 8 recording sites, without a length dependent pattern). The one participant who declined tilt table test also declined QSART testing for a non-medical reason.

### Data analysis

If the distribution of a variable was approximately normal by the Shapiro-Wilk normality test, the null hypothesis was examined with the 2-tailed Student's *t*-test. If the distribution failed the normality test we used the nonparametric Mann-Whitney *U*-test. Multiple comparisons were corrected using the Holm-Bonferroni method to control Type I error rate at no more than 5% (Holm, 1979). We used Spearman rank correlation method to assess the association between 2 continuous variables with SigmaPlot 11.2 software package (San Jose, CA; 2008).

## Results

### Participant characteristics

Table 1 shows participant demographics and autonomic symptoms obtained through on-site neurological evaluation at the time of clinical study. Ill participants appeared to have a higher body mass index (BMI) on average than that of Control participants. In the Ill group 2 participants had mild Hypertension while being treated with either Hydrochlorothiazide or Amlodipine, 1 metabolic syndrome, 1 with history of ulcerative colitis and hypothyroidism on Synthroid. In the Control group 1 had mild Hypertension on diet control and 1 coronary artery disease on Aspirin.

**Table 1:** **Demographics and autonomic symptoms**.

**Group**	**Ill (*n* = 16)**	**Control (*n* = 12)**
Age (mean ± s.e.m.)	48.3 ± 1.4	48.1 ± 2.0
Sex(M/F)	13/3	11/1
BMI (mean ± s.e.m.)	33.8 ± 2.2	28.2 ± 1.4
Post-exertion fatigue	16	0
Blurred vision	11	1
Weakness	10	0
Anxiety	8	1
Tremor	7	1
Dizziness	7	1
Abdominal pain	6	2
Dry eye/dry mouth	6	1
Distal paresthesia	5	0
Sexual dysfunction	4	2
Syncope	3	1
Night diarrhea	3	0

### Baseline autonomic function

Table 2 shows baseline cardiovascular measures. There was no significant group difference of blood pressures between the Ill and Control at supine, active standing, and 70° HUT. The heart rates at 5 min supine, 3 min standing, and 5 min 70° HUT were higher in the Ill group than Control. The absolute heart rate increment by HUT was not different between the 2 groups. HRV_DB_ (E/I ratio) and HRV_VM_ were not significantly different between the 2 groups.

**Table 2:** **Baseline autonomic function**.

	**Ill (*n* = 16)**	**Control (*n* = 12)**	***P*-Value**
Supine systolic BP	129.4±1.9	132.3±2.5	0.377
Supine diastolic BP	76.8±1.2	77.4±1.7	0.661
Supine HR	80.1±2.2	63.6±2.8	<0.001^*^
Standing systolic BP	125.3±2.5	129.2±2.4	0.277
Standing diastolic BP	80.1±1.1	83.2±2.6	0.530
Standing HR	93.3±3.8	75.4±3.7	0.003^*^
Standing HR increase	15.1±2.3	11.3±2.4	0.257
Standing systolic BP fall	−6.2±3.3	−4.9±2.0	0.764
Standing diastolic BP increase	4.1±1.3	5.9±1.8	0.400
HUT systolic BP fall	−11.9±3.1	−3.6±4.7	0.139
HUT diastolic BP fall	−11.2±2.7	−13.8±3.2	0.678
HUT maximum HR	94.3±3.2	83.2±4.6	0.053
HUT HR increase	18.0±2.6	19.8±3.6	0.673
E/I ratio	1.38±0.04	1.33±0.03	0.662
Valsalva ratio	1.63±0.03	1.89±0.08	0.078

Test results expressed as means ± s.e.m; HR, heart rate (beats/min); HUT, head-up tilt (*n* = 15 in the Ill group) for 5 min; BP, blood pressure; E/I ratio, expiratory/inspiratory ratio;

* Values remain significant after Holm-Bonferroni correction for multiple comparison.

### Diagnostic profiles

Among 16 participants in the Ill group we diagnosed 1 participant with Orthostatic Hypotension, 1 POTS, 2 SFN, and 1 length-dependent peripheral neuropathy affecting both large and small fiber (Table 3). We speculated underlying pathogenesis for neurological disorders based on past or current co-morbidities of each participant. Focal neuropathies of mild severity were noted in 4 participants among Ill group: 2 bilateral CTS, 1 unilateral CTS and contralateral ulnar neuropathy, and 1 unilateral CTS. Two Controls had mild focal neuropathy: 1 bilateral CTS and 1 non-localizing ulnar neuropathy. Fourteen Ill participants and 3 Controls who received needle EMG showed neither electrophysiological evidences of muscle disorders nor neuromuscular transmission disorders.

**Table 3:** **Diagnostic profile among Ill group with post-exertional fatigue**.

**Case#**	**Dx**	**Age/sex**	**Exam**	**HUT**	***E/I***	***VR***	**QSART**	**Sural SNAP**		**Speculated pathogenesis**
								**Left**	**Right**	
1	OH	51/F	Orthostatic dizziness	Presyncope^Δ^	1.47	1.56	Patchy reduction	ND	19.6	Idiopathic
2	POTS	58/M	Orthostatic headache	Orthostatic headache^$^	1.09	1.47	Patchy reduction	14	12.5	Metabolic
3	SFN	42/M	Distal dysthesia	Normal	1.60	1.77	Length- dependent reduction	15.1	7.4	Idiopathic
4	SFN	41/M	Distal dysthesia	Orthostatic dizziness^*^	1.24	1.63	Length- dependent reduction	10.5	9.4	Idiopathic
5	PN	49/F	Distal dysthesia	Normal	1.33	1.60	Length- dependent reduction	4.3	4.1	Autoimmune

Dx, diagnosis; HUT, head-up tilt for 20 min; E/I, expiratory/inspiratory ratio; VR, Valsalva ratio; QSART, quantitative sudomotor axon reflex test; Sural SNAP, sural sensory nerve action potential (B–point); OH, orthostatic hypotension; ND, not done; POTS, postural orthostatic tachycardia syndrome; SFN, small fiber neuropathy; PN, peripheral neuropathy;

Δ HUT terminated at 6 min due to systolic blood pressure drop >27 mmHg;

$ Heart rate increment >30 beats/min occurred within 9 min and persisted to the end of HUT;

* HUT terminated at 12 min due to severe dizziness.

### Large and small fiber nerve variables

There were no significant group differences between Ill and Control in large fiber nerve variables measured on sural sensory and peroneal motor nerves (Table 4). Results were calculated from one peroneal motor and one sural nerve from each of the 15 Ill participants who also had QSART as well as each of the 12 Controls. Sural SNAP responses, and amplitude as well as distal latency of compound muscle action potentials were within the normal limits of the reference values of our laboratory. Although the averaged sweat volume (ul/cm^2^) from the feet by QSART was not significantly different between the 2 groups after Holm-Bonferroni correction for multiple comparisons, it was notable that the Foot/Leg ratio also revealed a trend of length-dependent pattern of reduced sudomotor response among Ill participants.

**Table 4:** **Nerve conduction and sudomotor variables**.

	**Sural SNAP (mean ± s.e.m.)**		**Peroneal CMAP (mean ± s.e.m.)**		**QSART (mean ± s.e.m.)**		
	**Amplitude (μ V)**	**Latency (ms)**	**Amplitude (mV)**	**Distal latency (ms)**	**Proximal leg (μ l)**	**Foot (μ l)**	**Foot/leg ratio**
Ill (*n* = 15)	12.3 ± 1.9	3.4 ± 0.6	4.3 ± 0.5	5.2 ± 0.8	2.85 ± 0.33	1.45 ± 0.22	0.53 ± 0.06
Control (*n* = 12)	11.9 ± 2.1	3.5 ± 0.4	5.4 ± 0.6	4.9 ± 0.5	3.86 ± 0.46	2.88 ± 0.45	0.70 ± 0.07
*P*-value	0.598	0.235	0.178	0.462	0.077	0.011	0.097

Sural SNAP, sural sensory nerve action potential measured at B point; Peroneal CMAP, peroneal nerve compound muscle action potential; QSART, quantitative sudomotor axon reflex test.

### Quantitative sensory testing

**Figure 1:**
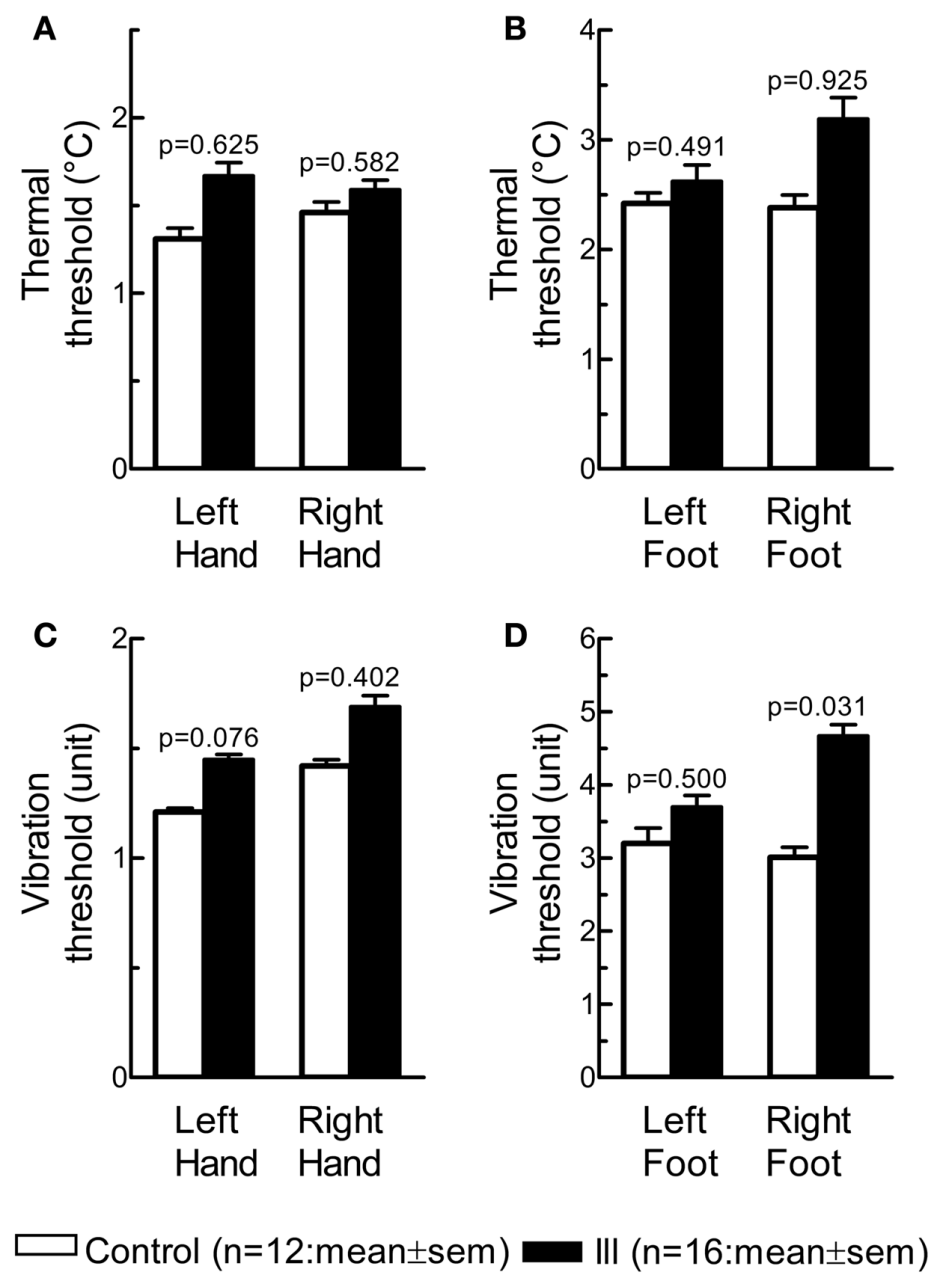
**Quantitative sensory testing.** Thermal threshold (°C) on hands and feet, shown as vertical bars **(A,B)**, is a measure of distal small somatic nerve fiber function; and vibration threshold **(C,D)** a measure of large nerve fiber function.

The *p*-value was calculated from each of the 8 independent Mann-Whitney *U*-tests. There were no significant group differences for thermal (Figures 1A,B) and vibration (Figures 1C,D) threshold on hands and feet after Holm-Bonferroni correction for multiple comparison.

### Composite autonomic scoring scale

The CASS was significantly higher among Ill group [(*n* = 15); 95% CI of mean: 1.72–2.67; Table 5] compared to Control [(*n* = 12); 95% CI of mean: 0.58–1.59; *p* = 0.004, Mann-Whitney *U*-test]. Among the Ill group the CASS was associated with post-exertional Fatigue Severity Score [(*n* = 15); Spearman's rank correlation coefficient: 0.571; *p* = 0.025].

**Table 5:** **Composite Autonomic Scoring Scale. Test results expressed as means ± s.e.m**.

	**Ill (*n* = 15)**	**Control (*n* = 12)**	***P*-value**
Total CASS	2.20 ± 0.22	1.08 ± 0.23	0.004
Sudomotor	1.20 ± 0.20	0.67 ± 0.18	0.075
Cardiovagal	0.53 ± 0.17	0.33 ± 0.14	0.440
Adrenergic	0.47 ± 0.13	0.17 ± 0.11	0.114

## Discussion

Patients with chronic unexplained fatigue, dizziness, and tremor may have a neuro-pathogenesis after a careful objective neurophysiologic assessment, despite such complaints often being mistakenly regarded as psychological in origin (Schondorf et al., 1999; Thieben et al., 2007). Our study participants had many years of unexplained complaints of post-exertional fatigue after deployment to Gulf theater. Due to the chronicity of the illness, ill participants had already sought medical attention before being recruited into our study, thus it was not surprising that commonly suspected neuromuscular disorders such as myopathy, myasthenia gravis, or large fiber neuropathy were not significantly associated with the unexplained complaints in our and other's studies. Our effort to study the autonomic dysfunction for unexplained multi-symptoms in ill GW veterans is in line with the research goals of others (Sharief et al., 2002; Haley et al., 2004; Khan et al., 2004; Stein et al., 2004). Our study, however, is unique in the following 2 aspects. First, we have studied not only the group difference between the Ill and Control in a prospective structured fashion from a population based sample but also attempted to characterize individual participants clinically. Secondly, in the context of history taking and physical examination on each individual participant, we intended to identify clinically definable autonomic disorders through objective testing on large and small somatic nerve function by NCS and QST, cardiovagal function by HRV_DB_ and HRV_VM_, adrenergic function by tilt table testing, and sympathetic postganglionic sudomotor function by QSART which was also used for fulfilling the diagnostic criteria of small fiber neuropathy in this study.

We found significantly higher CASS among the Ill participants compared to Controls. Among 3 domains of CASS the group difference of sudomotor subscore appeared to approach the significance level. Ill participants as a group showed a trend of reduction of sweat volume on foot more than that of proximal sites by QSART compared to Controls, suggesting the possibility of a length-dependent small fiber dysfunction. It is possible that the preferentially dysfunctional postganglionic cholinergic fibers were contributing to the post-exertional fatigue in some Ill participants, though we cannot exclude chronic illness resulting in secondary postganglionic cholinergic fiber dysfunction because patchy sudomotor abnormalities were common among patients with autonomic dysregulation (Peltier et al., 2010). It is also possible that the cardiovagal and adrenergic CASS subscores collected on study day 2 or 3 among the Ill participants were underestimated values due to the repeated exposures to cardiovascular stress during the training or testing on prior study days (Reybrouck et al., 2000). We identified 4 Ill participants with definable autonomic disorders (2 idiopathic SFN, 1 orthostatic hypotension, and 1 POTS who also had a borderline low E/I ratio of 1.09) and 1 peripheral neuropathy with both large and small somatic fiber dysfunction. All these 5 Ill participants had sudomotor abnormalities by QSART. Based on these data we are reasoning that peripheral and visceral small nerve fibers may be the potential neurological substrates preferentially affected in some Ill participants with unexplained autonomic symptoms. Among the Ill participants the CASS was associated with self-reported post-exertional Fatigue Severity Score. If the noted overrepresentation of autonomic and somatic small fiber dysfunction among participants in our study can be confirmed in a larger clinical series, we suggest that autonomic nerves are potential therapeutic targets for intervention of some ill veterans' unexplained post-exertional fatigue.

Given the worse CASS score and definable autonomic disorders identifiable only among Ill participants compared to Controls, a higher baseline supine heart rate 80 beats/minute of our Ill group compared to 64 beats/minute of Controls suggests the involvement of neural mechanism for chronic fatigue (De Becker et al., 2000; Newton et al., 2011). In a group of 23 patients meeting the CDC criteria for the diagnosis of chronic fatigue syndrome it was noted that the patient group had both higher baseline heart rate and higher heart rate increment on upright tilt compared to controls, suggesting orthostatic intolerance as a main feature of autonomic nervous system involvement (Freeman and Komaroff, 1997). Our Ill participants do not meet the CDC chronic fatigue syndrome criteria, but can be classified into idiopathic chronic fatigue (Fukuda et al., 1994). In agreement with the above mentioned study ours also showed a higher baseline heart rate among the Ill participants compared to Controls, suggesting a role of cardiovascular deconditioning related to the sedentary life style. The lack of group difference of heart rate increment on upright tilt in our study, which is inconsistent with classical deconditioning measured by passive HUT, may partly be explained by: (a) that the military veterans are physically healthier than age-matched civilians and 5 min HUT is not sufficient to cause measurable group difference, and/or (b) that factors in addition to deconditioning also play roles in the initiation or maintenance of orthostatic intolerance.

The Ill participants had a higher average BMI than Controls. It was estimated that one standard deviation change in BMI resulted in a decrease in the E/I ratio of 0.010–0.014 and a decrease in the HR_Max−Min_ of 1.56–2.39 beats/min (Freeman et al., 1995). Given the mean E/I ratio of the Ill group in our study was 1.38, not different from 1.33 of Control, it is unlikely that BMI had significant influence on the cardiovagal parameters. When 4 participants with highest BMI were removed from the Ill group for calculation, the significance of differences (*p* < 0.05) remained for heart rates at baseline supine and standing as well as CASS calculation between the Ill and Control. The noted identifiable autonomic disorders and higher CASS among the Ill participants compared to Controls argue for a process of deleterious interplay among autonomic dysfunction, high BMI, and fatigue. It is conceivable that environmental factors such as endocrine disrupting compounds exposed during deployment or repetitively administered multiple vaccinations in a short period, might involve the development of obesity or autonomic A-delta/C fiber dysfunction that directly related to the development of post-exertional fatigue. On the other hand, it is also plausible that the mechanism leading to autonomic dysfunction might be independent of the biological or biopsychological process that related to post-exertional fatigue. Metabolic disturbance such as pre-diabetes, however, is one of the commonly implicated causes for visceral or small somatic fiber neuropathy in general population. Given the insidious onset and chronic course of autonomic symptoms among most Ill participants, one could speculate that underlying metabolic disturbance may be etiologically associated with autonomic dysfunction, high BMI, and fatigue.

Because the study participant numbers were small, and the study was not conducted in a blind fashion, the interpretation of our findings concerning large numbers of deployed veterans is limited. Variables that were not analyzed in this report such as anxiety and undetected depression may be related to various levels of self-reported post-exertional fatigue, confounding the interpretative validity of our findings and limiting the comparability between this study and future studies by others with a similar design. It remains unclear if our findings represent a cause, a consequence or an unrelated co-morbidity of the primary illness being studied. The lack of significant group differences of thermal threshold by QST, a surrogate for small somatic fiber function, between the Ill and Control, may be due to the insensitivity of QST we have applied. Skin biopsy may become an alternative tool in future studies.

In conclusion, we have identified definable autonomic disorders and shown measurable autonomic dysfunction among a group of GW veterans who had persistent, unexplained post-exertional fatigue, among other autonomic symptoms, after military deployment. Our observation paves the way for future studies using non-deployed controls to investigate the effects of environmental exposures or stress during the deployment on the autonomic neuromuscular system at a detailed physiological level. Our study has practical pertinence to the clinical care of deployed GW veterans. An ill veteran with persistent, unexplained post-exertional fatigue that has no anticipated responses to conventional treatment may require objective evaluation for autonomic function.

### Conflict of interest statement

The authors declare that the research was conducted in the absence of any commercial or financial relationships that could be construed as a potential conflict of interest.
